# Supplementary material for: Bound for the nucleus: defining the molecular principles of cargo selection by importin 9
Source: bioRxiv. 2026 May 14:2026.05.12.724649. Preprint. [Version 1] doi: 10.64898/2026.05.12.724649 (PMC13192796; doi:10.64898/2026.05.12.724649)

bioRxiv preprint doi: https://doi.org/10.64898/2026.05.12.724649; this version posted May 14, 2026. The copyright holder for this preprint (which was not certified by peer review) is the author/funder, who has granted bioRxiv a license to display the preprint in perpetuity. It is made available under a CC-BY 4.0 International license.

Supplemental Table 1

PAN-GO Biological Process enrichment analysis of IPO9 cargo (PANTHER, FDR <0.05)

GO Term	GO ID	Ref	Hits	Fold Enrichment	FDR
Proteasome & Protein Degradation					
Proteasome-mediated ubiquitin-dependent protein catabolism	GO:0043161	244	13	14.06	2.55E-08
Modification-dependent protein catabolic process	GO:0019941	379	14	9.75	1.64E-07
Ubiquitin-dependent protein catabolic process	GO:0006511	370	13	9.27	8.79E-07
Proteolysis involved in protein catabolic process	GO:0051603	452	15	8.76	1.80E-07
Protein catabolic process	GO:0030163	467	15	8.47	1.77E-07
Proteasomal protein catabolic process	GO:0010498	299	13	11.47	1.62E-07
Chromatin & DNA					
Heterochromatin formation	GO:0031507	76	4	13.89	4.03E-02
DNA-templated transcription	GO:0006351	140	5	9.42	4.01E-02
Chromatin organization	GO:0006325	266	9	8.93	2.67E-04
Chromatin remodeling	GO:0006338	222	7	8.32	5.65E-03
RNA Processing & Metabolism					
mRNA alternative polyadenylation	GO:0110104	2	2	>100	3.91E-03
rRNA metabolic process	GO:0016072	147	5	8.97	4.74E-02
mRNA processing	GO:0006397	181	6	8.75	1.54E-02
mRNA metabolic process	GO:0016071	255	7	7.24	1.27E-02
RNA biosynthetic process	GO:0032774	619	14	5.97	3.66E-05
RNA metabolic process	GO:0016070	742	15	5.33	4.76E-05
RNA processing	GO:0006396	479	9	4.96	1.76E-02
Broader Metabolic Processes					
Nucleic acid biosynthetic process	GO:0141187	639	14	5.78	4.73E-05
Nucleobase-containing compound biosynthetic process	GO:0034654	764	14	4.83	3.11E-04
Nucleic acid metabolic process	GO:0090304	1060	18	4.48	3.37E-05
Macromolecule catabolic process	GO:0009057	581	16	7.27	3.46E-07
Catabolic process	GO:0009056	1007	16	4.19	3.25E-04
Gene expression	GO:0010467	1159	17	3.87	3.84E-04
Protein metabolic process	GO:0019538	1409	20	3.75	7.76E-05
Macromolecule metabolic process	GO:0043170	2499	37	3.91	1.10E-10
Nucleobase-containing compound metabolic process	GO:0006139	1318	18	3.60	4.69E-04

Ref = number of genes in reference list; Hits = number of genes in uploaded list
FDR = false discovery rate corrected p-value (Fisher's exact test)

bioRxiv preprint doi: https://doi.org/10.64898/2026.05.12.724649; this version posted May 14, 2026. The copyright holder for this preprint (which was not certified by peer review) is the author/funder, who has granted bioRxiv a license to display the preprint in perpetuity. It is made available under a CC-BY 4.0 International license.

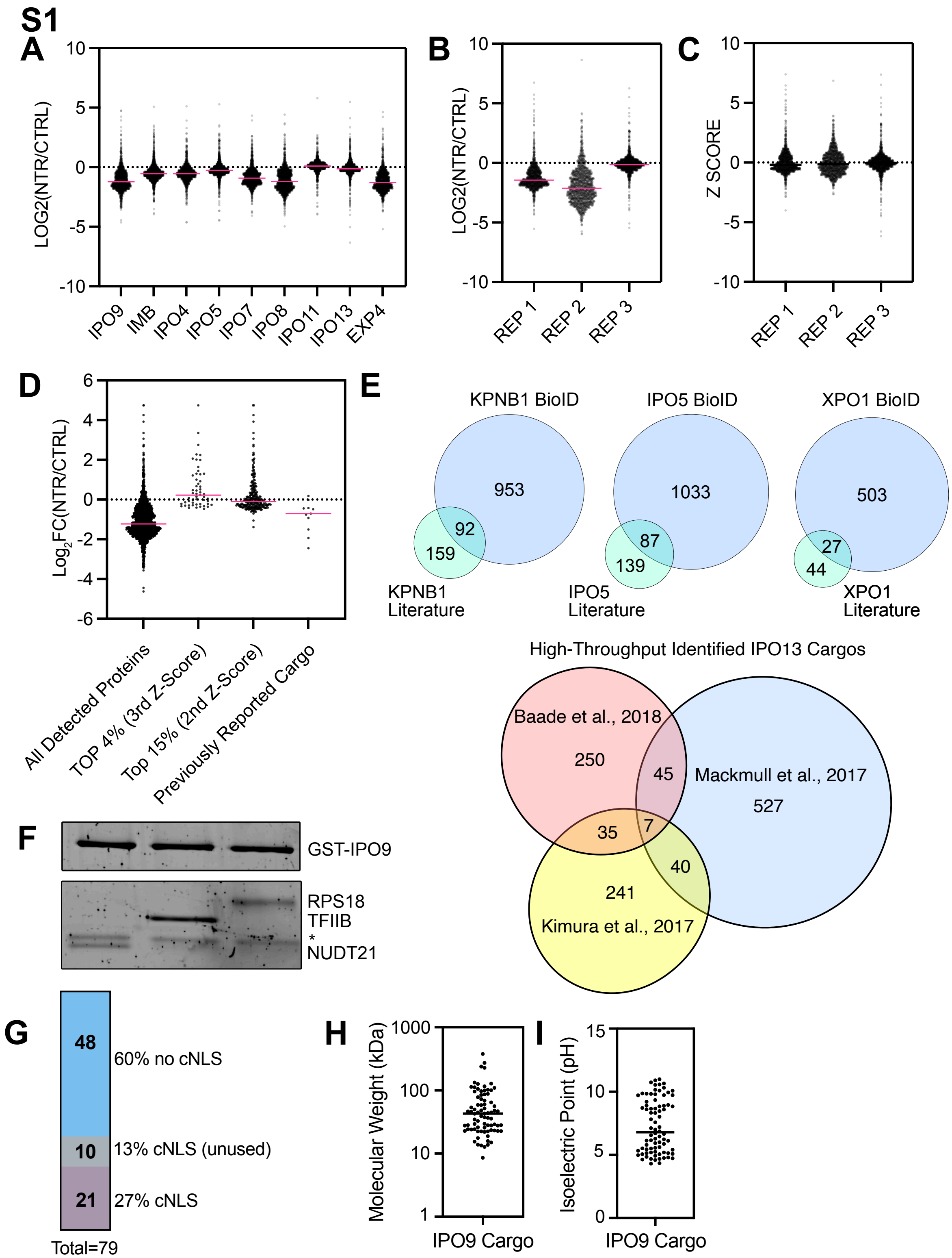

bioRxiv preprint doi: https://doi.org/10.64898/2026.05.12.724649; this version posted May 14, 2026. The copyright holder for this preprint (which was not certified by peer review) is the author/funder, who has granted bioRxiv a license to display the preprint in perpetuity. It is made available under a CC-BY 4.0 International license.

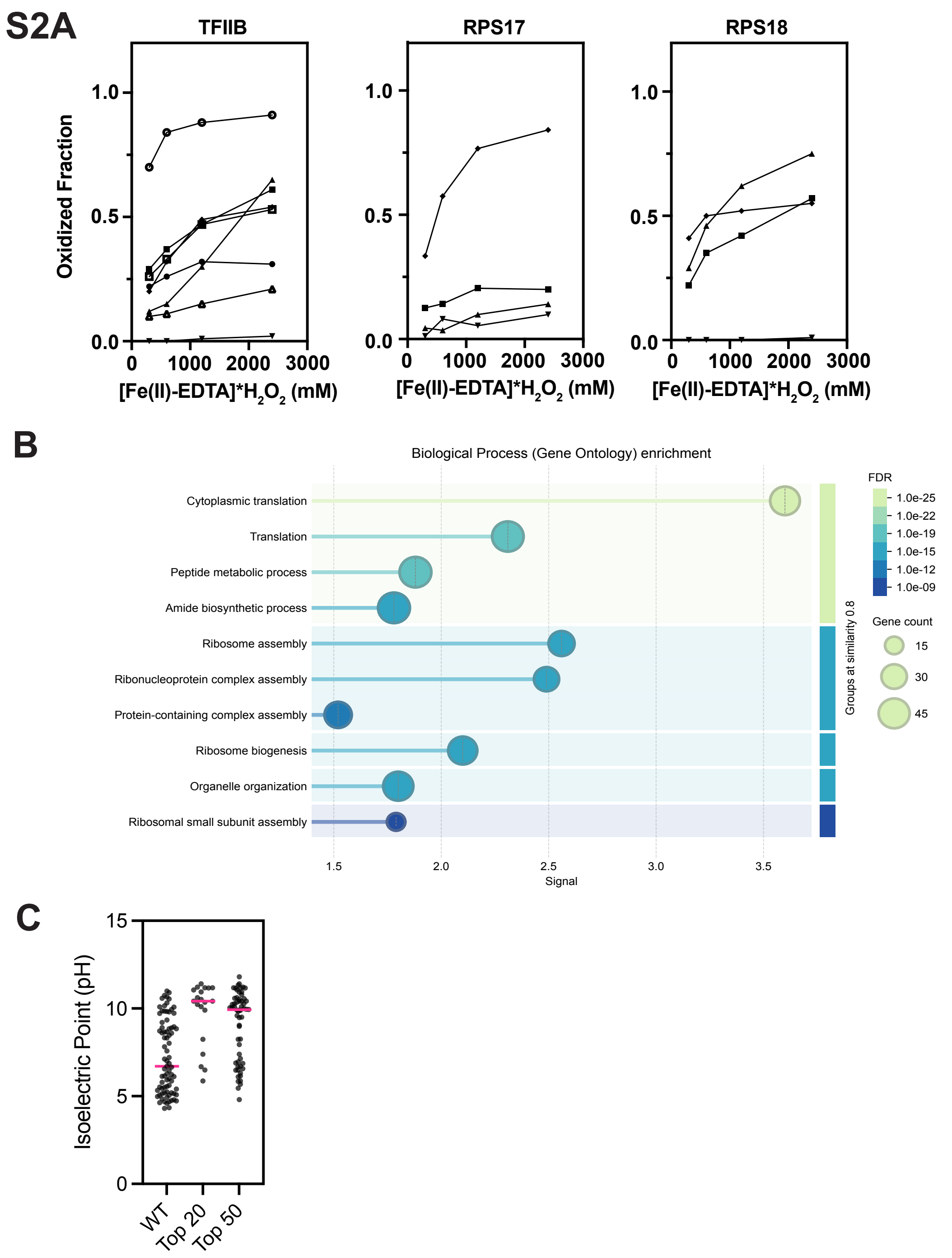

bioRxiv preprint doi: https://doi.org/10.64898/2026.05.12.724649; this version posted May 14, 2026. The copyright holder for this preprint (which was not certified by peer review) is the author/funder, who has granted bioRxiv a license to display the preprint in perpetuity. It is made available under a CC-BY 4.0 International license.

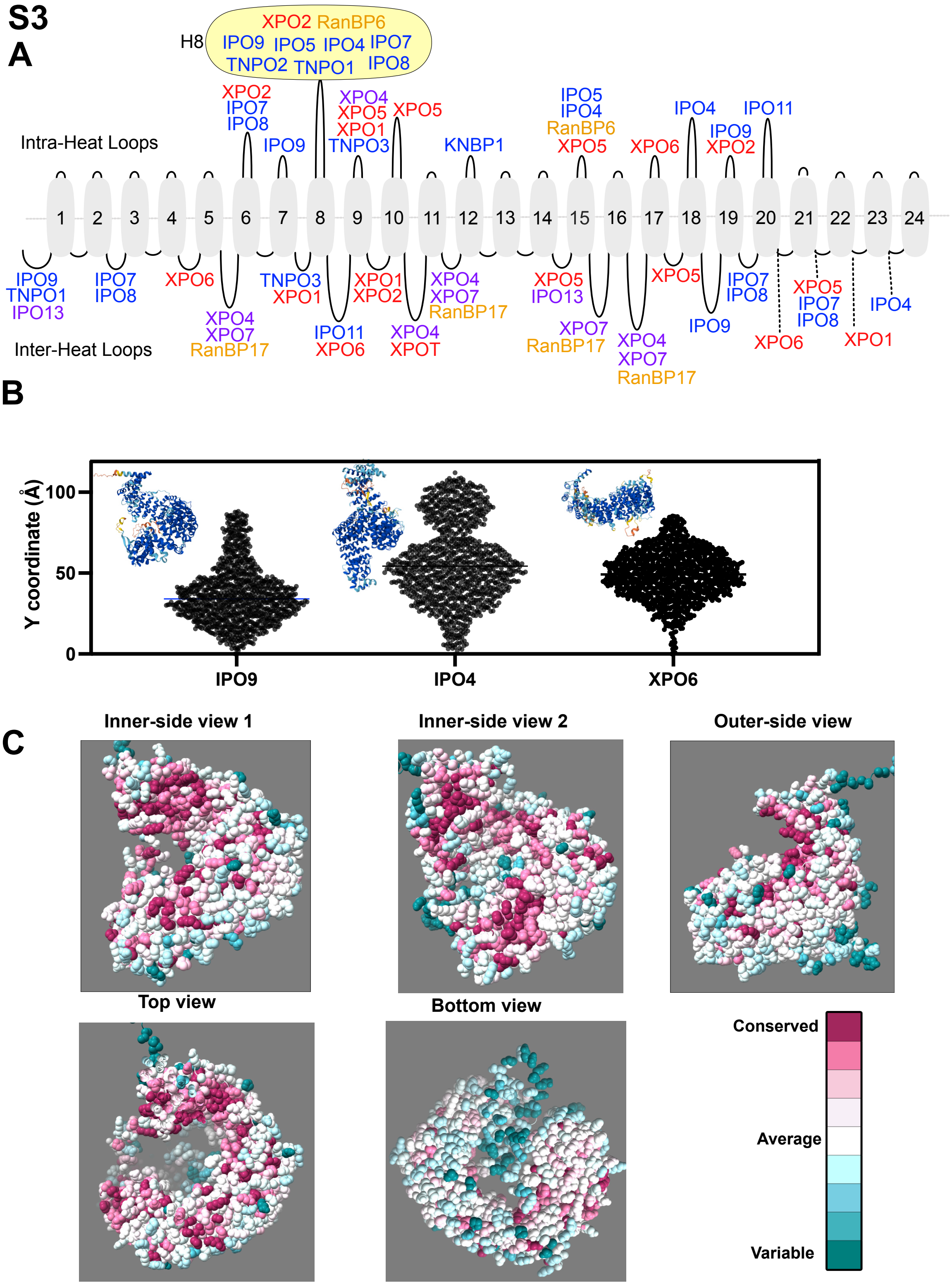

bioRxiv preprint doi: https://doi.org/10.64898/2026.05.12.724649; this version posted May 14, 2026. The copyright holder for this preprint (which was not certified by peer review) is the author/funder, who has granted bioRxiv a license to display the preprint in perpetuity. It is made available under a CC-BY 4.0 International license.

S4A

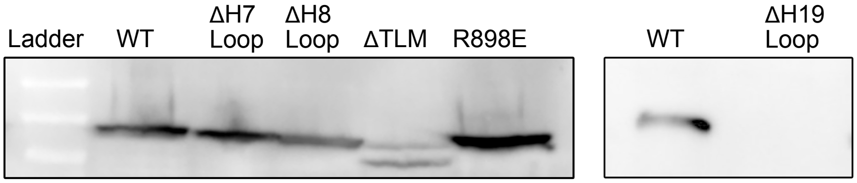

bioRxiv preprint doi: https://doi.org/10.64898/2026.05.12.724649; this version posted May 14, 2026. The copyright holder for this preprint (which was not certified by peer review) is the author/funder, who has granted bioRxiv a license to display the preprint in perpetuity. It is made available under a CC-BY 4.0 International license.

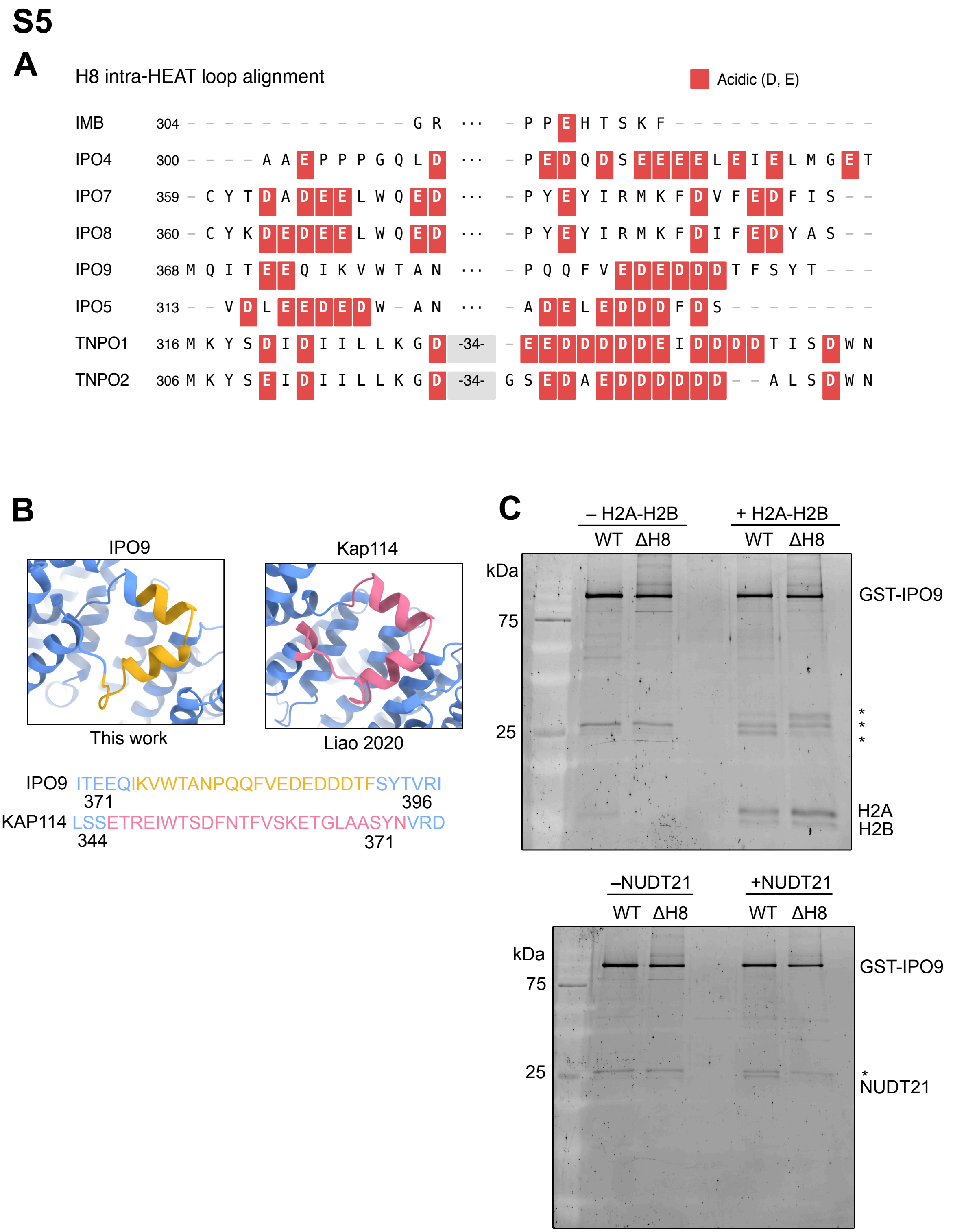

bioRxiv preprint doi: https://doi.org/10.64898/2026.05.12.724649; this version posted May 14, 2026. The copyright holder for this preprint (which was not certified by peer review) is the author/funder, who has granted bioRxiv a license to display the preprint in perpetuity. It is made available under a CC-BY 4.0 International license.

S6

A

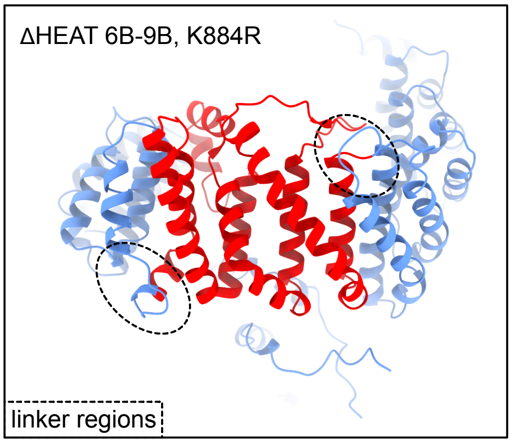

ΔTLM

B

Unchanged in ΔTLM (vs WT)		
DDB1	PSMA5	PSMB7
EIF1AX	PSMA6	RAN
KCTD9	PSMA7	RANBP1
PPIB	PSMB1	RPS17
PRMT5	PSMB2	RPS27A
PSMA1	PSMB3	TRIM21
PSMA2	PSMB4	TFIIB
PSMA3	PSMB5	YBOX1
PSMA4	PSMB6	YBOX3

C

Lost in ΔTLM (vs WT)		Gained in ΔTLM (vs WT)	
CCDC130	NUDT21	CLNS1A	RPL7A
CPSF6	PELO	DDB1	RPLP0
CUL2	POGK	EEF2	RPS14
FMC1	PSAT1	EIF3A	RPS2
H2A	PTOV1	EIF3I	RPS3
H2B	RPS5	IRS4	RPS3A
H2AFY	RPS7	NAP1L1	RPS4X
H2AFZ	RPS18	PA2G4	RPS6
KLDC2	YWHAB	PRSS1	RPS8
MAGED2	YWHAE	RACK1	RPS25
MRPL39	YWHAQ	RPL12	RPSA
NFYA		RPL13	SEPBP1
		RPL15	STIP1
		RPL6	ST13P4

Also lost in
ΔH8
R898E

Also gained in
ΔH7
ΔH8
R898E

D

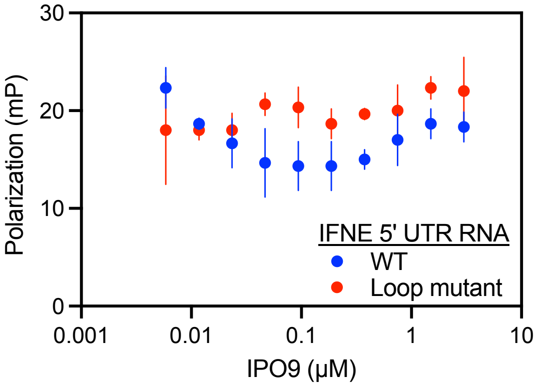

bioRxiv preprint doi: https://doi.org/10.64898/2026.05.12.724649; this version posted May 14, 2026. The copyright holder for this preprint (which was not certified by peer review) is the author/funder, who has granted bioRxiv a license to display the preprint in perpetuity. It is made available under a CC-BY 4.0 International license.

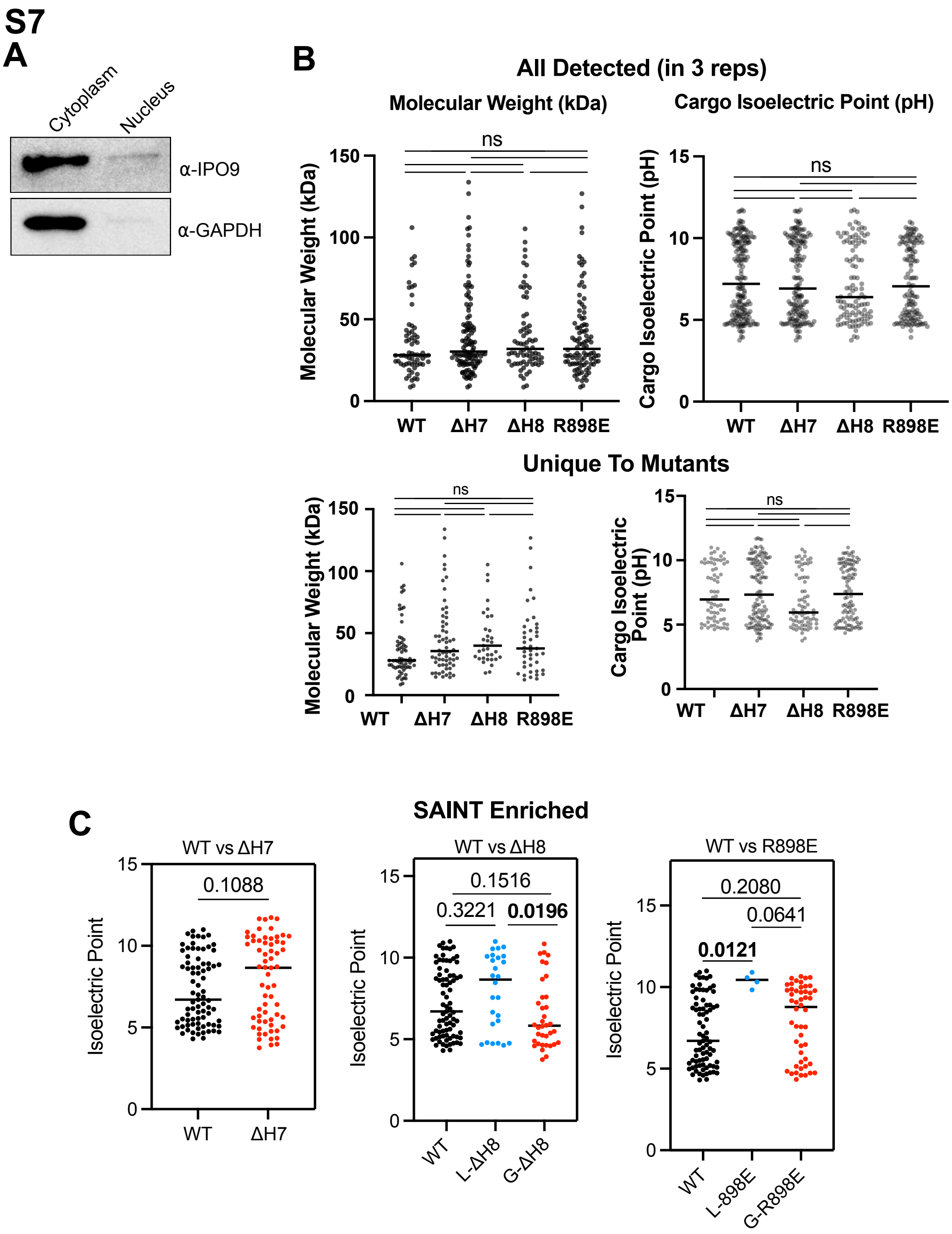

Supplement: 1 — Supplemental Table 1. PANTHER GO term analysis for cargos enriched SAINT >0.9. Supplemental Figure 1. Comparison to previous importin•cargo interaction data and further investigation into identified IPO9 cargo characteristics. (A) Graph detailing the Kimura 2017 group’s log2 fold-change in all cargos measured for each importin. NTR means nuclear transport receptor, aka karyopherins. Note variation from baseline (0 fold change) for IPO9, IPO7, IPO8, and EXP4/XPO4. This variation may be expected for XPO4 as it is a biportin, so one would expect less nuclear signals of some cargoes. (B) Log2 fold-change for IPO9 experiment by Kimura 2017 group broken out by replicate. Large variation between replicates. (C) Transformation of Log2 fold-change values into Z-scores masks the variation seen in the raw data. (D) When looking of the Log2 Fold changes of cargo identified as significant (top 4% 3rd z-score n=53, top 15% 2nd z-score n=254), one can see that many of these cargos are not enriched in the +IPO9 samples. Import scores of previously reported cargo are similarly variable. (E) Recapitulation of Venn diagrams from Mackmull 2018 paper showing overlap of identified cargo with existing dataset. (F) SYPRO Ruby stained 10% SDS-PAGE gel depicting putative cargo pulldowns with highly purified recombinant factors: GST-IPO9 and NUDT21, TFIIB, or RPS18 (*corresponds to nonspecific protein present in GST-IPO9 protein preparation). (G) Plot depicting the fraction of cargo that have mono- or bi- partite NLSs present in their amino acid sequence by NLSstradamus (46). (H) Chart depicting molecular weight of identified cargos (78). (I) Chart depicting isoelectric point of identified cargo. IPO9, importin 9. Supplemental Figure 2. Ox-FT identified peptides of cargo proteins. (A) Peptide oxidation fraction for three different cargo proteins. Supplemental Figure 3. Radius and pitch of importins is variable despite HEAT repeat conservation. (A) Depictions of loops of all karyopherins sprea [file NIHPP2026.05.12.724649V1-supplement-1.pdf]
